# Expression of Transgenes Targeted to the *Gt(ROSA)26Sor* Locus Is Orientation Dependent

**DOI:** 10.1371/journal.pone.0000004

**Published:** 2006-12-20

**Authors:** Douglas Strathdee, Helen Ibbotson, Seth G. N. Grant

**Affiliations:** 1 The Wellcome Trust Sanger Institute, Wellcome Trust Genome Campus Hinxton, Cambridge, United Kingdom; 2 Centre for Neuroscience Research, University of Edinburgh Edinburgh, Scotland; The Babraham Institute, United Kingdom

## Abstract

**Background:**

Targeting transgenes to a chosen location in the genome has a number of advantages. A single copy of the DNA construct can be inserted by targeting into regions of chromatin that allow the desired developmental and tissue-specific expression of the transgene.

**Methodology:**

In order to develop a reliable system for reproducibly expressing trangenes it was decided to insert constructs at the *Gt(ROSA)26Sor* locus. A cytomegalovirus (CMV) promoter was used to drive expression of the Tetracycline (tet) transcriptional activator, rtTA2^s^-M2, and test the effectiveness of using the *ROSA26* locus to allow transgene expression. The tet operator construct was inserted into one allele of *ROSA26* and a tet responder construct controlling expression of EGFP was inserted into the other allele.

**Conclusions:**

Expression of the targeted transgenes was shown to be affected by both the presence of selectable marker cassettes and by the orientation of the transgenes with respect to the endogenous *ROSA26* promoter. These results suggest that transcriptional interference from the endogenous gene promoter or from promoters in the selectable marker cassettes may be affecting transgene expression at the locus. Additionally we have been able to determine the optimal orientation for transgene expression at the *ROSA26* locus.

## Introduction

Spatial and temporal control of gene expression represents an extremely powerful tool for the analysis of gene function and the events underlying complex biological processes such as embryonic development and cognitive function [1], [2].

One method of reversibly controlling gene activity is to regulate its transcription [3]. The transcription control system based on elements of the tetracycline (tet) resistance operon of E. coli have been widely used to control gene expression in mammalian cells. One of the key components of the tet system is the tetracycline-controlled transactivator (tTA), a fusion protein between the repressor of the Tn10 tet resistance operon of Escherichia coli and a C-terminal portion of VP16 that contains domains capable of activating transcription [4]. tTA will activate transcription from a suitably engineered minimal promoter by binding to an array of tet operator sequences positioned upstream. Random mutagenesis of TetR generated a new transactivator (rtTA), which binds and transactivates gene expression in the presence of doxycycline (dox) [5]. Improved versions of rtTA have been developed to give tighter gene expression, increased sensitivity towards the inducer, enhanced stability and expression in mammalian cells, and more uniform transgene expression in the induced cells [6]–[8].

Introduced transgenes are frequently susceptible to gene silencing [9]. Although the mechanism for this process is poorly understood, both the integration site and the variable copy number at the integration site can influence the expression level [9]–[11]. This is often seen as progressive silencing of gene expression initially resulting in mosaic expression levels and often resulting in complete shutdown of transgene expression [12].

As randomly integrated transgenes are susceptible to gene silencing, targeting transgenes to a chosen location in the mouse genome has a number of advantages [13], [14]. Firstly the integration site can be chosen to allow insertion of the transgene into a region of chromatin favourable for expression and that avoids an undesirable insertional mutagenesis. Additionally only a single copy is be introduced which avoids problems associated with a large multicopy array.

The *Gt(ROSA)26Sor* locus (ROSA26) was first described as a gene trap which is ubiquitously expressed in mouse embryos [15]. As the ROSA26 locus is active in most cells any promoter inserted into the locus should not be restricted in its expression by unfavourable chromatin configurations. This locus has been widely used for expressing endogenous sequences, often reporter genes usually from the endogenous promoter [16], [17]. The promoter from the ROSA26 locus has been used to drive widespread expression of marker genes in transgenic rats and mice [18]. The ROSA26 promoter has also been used to express the tTA and rtTA successfully [19]–[21]. Other studies have shown that targeting tissue-specific promoters, including BAC sequences, to specific genomic locations leads to its expression in the appropriate tissue and cell-specific pattern [22]–[26].

In order to test if the ROSA26 locus was and if the local chromatin structure would effect transgene expression at the locus constructs expressing the rtTA and a reporter gene downstream of the tetracycline-responsive element (TRE) were targeted into ROSA26 locus in both orientations. Expression was found to be dependent on both the orientation of the transgene and the presence of an adjacent selectable marker.

## Results

### Targeting strategy for introducing to ROSA26 locus

**Figure 1:**
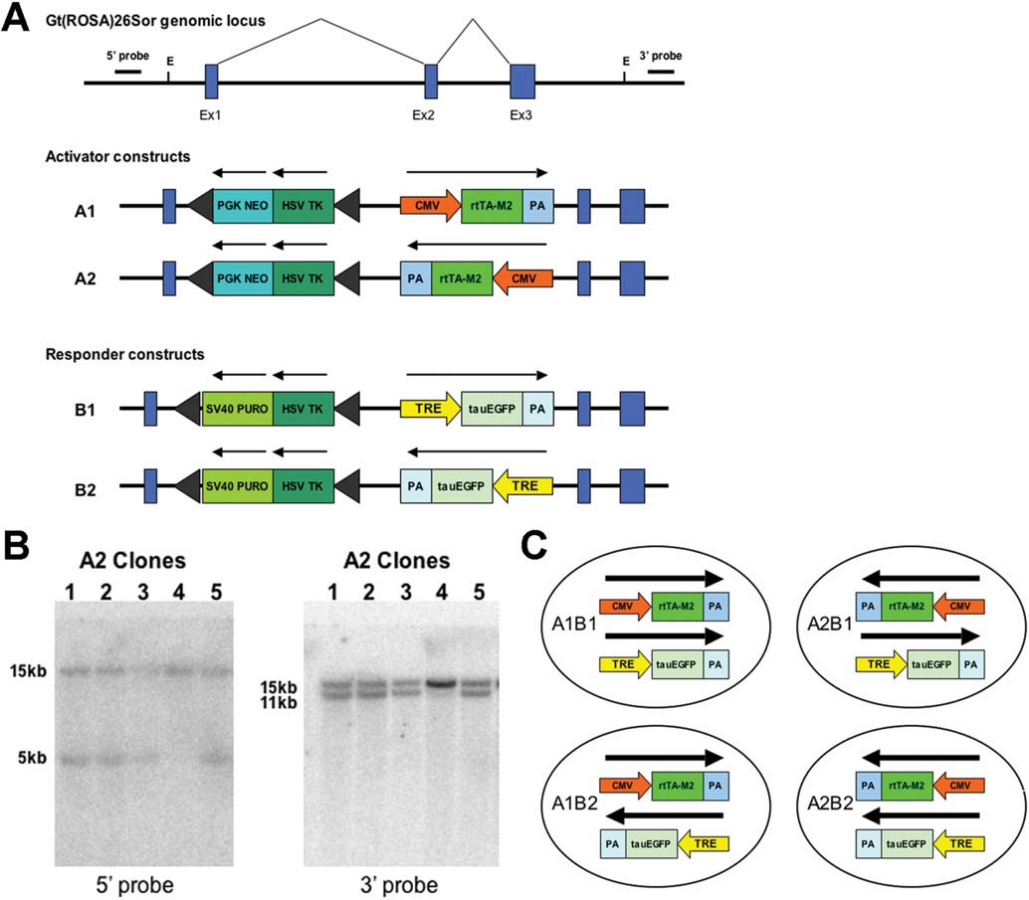
Targeting strategy for introducing to ROSA26locus. (**A**). Diagram of targeting constructs to introduce Dox responsive transgenes into locus. A1 and A2 introduce the same activator transgene in opposite orientations. B1 and B2 introduce the same responder transgene in opposite orientations. (**B**) Four different cell lines were produced with the activator and responder transgenes in different orientations (**C**) Southern blots probed with 5′ and 3′ flanking probes produce the correct band sizes to demonstrate appropriate targeting of constructs.

Establishing dox-dependent gene expression requires two different transgenes, an activator and a responder transgene. Activator constructs were generated with a CMV promoter driving the rtTA2^s^-M2 variant of the tetracycline transcriptional activator. The CMV promoter was chosen as it works inefficiently in ES cells and should be sensitive to position effects [27]. The cassette was cloned into the targeting vector in both orientations to produce the A1 and A2 targeting constructs (Figure 1A). The responder construct contained an EGFP transgene under control of the tetracycline responder element. This again was cloned into the targeting vector in both orientations to produce the B1 and B2 targeting vectors.

Cell lines were generated by sequentially targeting HM1 ES cells with first the activator and then the responder construct. Clones of cells which had been electroporated with the activator targeting vectors were isolated by selection with G418. Correctly targeted cells were initially identified by PCR and subsequently confirmed by Southern blot (Figure 1B). Cell lines which had the activator transgene correctly inserted at the ROSA26 locus were then electroporated with the responder transgene and selected on puromycin and G418. Resistant colonies were then analysed for correct targeting of the responder transgene to the ROSA26 locus. Consequently four different cell lines were generated with the activator and responder transgenes in both orientations (Figure 1C).

### Dox regulated gene expression works appropriately at the ROSA26 locus

**Figure 2:**
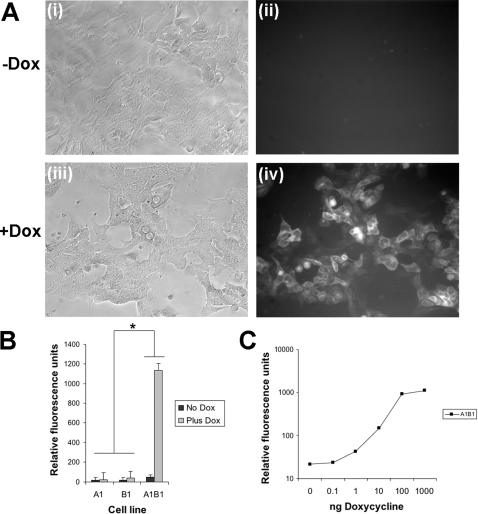
Dox regulated gene expression works appropriately at the ROSA26 locus. (**A**) EGFP expression induced in A1B1 cells by addition of dox to a final concentration of 1 µgml^−1^. (**i**) and (**iii**) Phase contrast. (**ii**) and (**iv**) EGFP fluorescence (**B**) EGFP expression in single targeted and double targeted cell lines in reponse to 1 µgml^−1^ dox. Induction of EGFP is only seen in the A1B1 cells (F(2,30) = 1167.61, p<0.0001). Each point is an average of three measurements each from two independently targeted cell lines. Error bars denote standard deviations. Asterisks indicate statistically significant differences. (**C**) Dose response curve EGFP expression in A1B1 cells in response to dox.

After targeting both the activator and responder constructs to the ROSA26 locus, EGFP expression was induced by treating the cells with doxycycline. No fluorescence was detected above background in single targeted cells, containing either the activator construct or responder construct alone (Figure 2B). This demonstrates that the responder construct has very low background activity when targeted into the ROSA26 locus.

Before addition of dox to the A1B1 double targeted cells containing both transgenes there again was no fluorescence detectable above background (Figure 2A and 2B). This demonstrates that there is very little leaky expression when both constructs are targeted to different alleles of the ROSA26 locus. Upon addition of dox to the medium, robust fluorescence was detected in the double targeted cells. This demonstrates that both transgenes are functioning appropriately when targeted to separate alleles of the locus. Furthermore EGFP expression induced by the activator transgene follows a similar dose response curve to that previously published (Figure 2C).

### Transgene expression level at ROSA26 is consistent between clones and dependent on transgene orientation

In order to test if the expression levels of the transgenes were influenced by the regulatory sequences at the ROSA26 locus both the activator (A) and responder (B) constructs were introduced into the locus in both orientations. Four double targeted cell lines were generated with different combinations of the two transgenes (Figure 1C).

**Figure 3:**
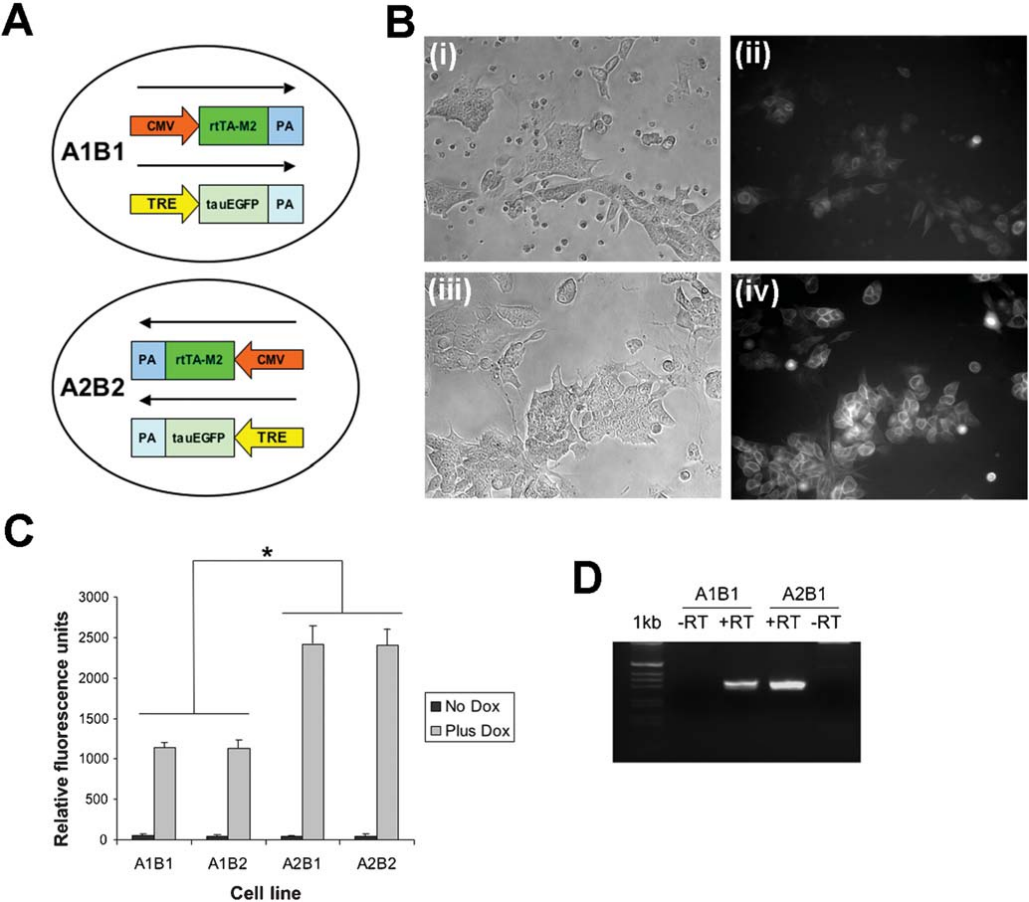
Expression level is dependent on orientation. (**A**) Diagram of orientation of constructs in cell lines. (**B**) A1B1 and A2B2 cells induced with doxycycline (**i**) A1B1 phase contrast (**ii**) A1B1 EGFP expression (**iii**) A2B2 phase contrast (**iv**) A2B2 EGFP expression (**C**) Graph of expression levels in induced and uninduced cell lines quantitated by fluorimetry. A2 cell lines have higher EGFP expression compared with the A1 cell lines (*F*(1,40) = 346.09, *p*<0.0001). Expression levels between the B1 and B2 cell lines were not significantly different (*F*(1,40) = 0.05, *p* = 0.8211). Each point is an average of three measurements each from two independently targeted cell lines. Error bars denote standard deviations. Asterisks indicate statistically significant differences. (**D**) Expression level of rtTA measured by RT-PCR.

Examples of the induction of EGFP expression in the A1B1 and A2B2 cell lines are shown (Figures 3A and 3B). Before administration of dox to the cells, none of the four cell lines showed any significant levels of EGFP expression (Figures 3B and 3C). This supports the conclusion that the level of background activity of the constructs is very low and that targeting the constructs to a region of active transcription does not initiate significant levels of background EGFP expression.

After administration of dox all four cell lines showed robust expression of EGFP (Figures 3B and 3C). When the expression was quantitated though, it was obvious that the transgenes that were in the opposite orientation to the ROSA26 promoter showed higher expression than those which were in the same orientation as the ROSA26 promoter (Figure 3C). This effect was more pronounced for the activator transgene than the responder transgene. The difference in level of induced EGFP expression correlated well with the difference in the level of expression of the rtTA transgene when measured by RT-PCR (Figure 3D). This suggests that the difference in the expression level between the different cell lines is mainly attributable to the level of rtTA expression driven by the CMV promoter.

The expression levels in at least two independently targeted cell lines were measured for each of the orientations described above. When the expression levels in these cell lines are averaged independently, the genetically identical cell lines vary by about 10–15% in induced expression of EGFP (data not shown).

### Expression level can be increased when selectable marker is removed

**Figure 4:**
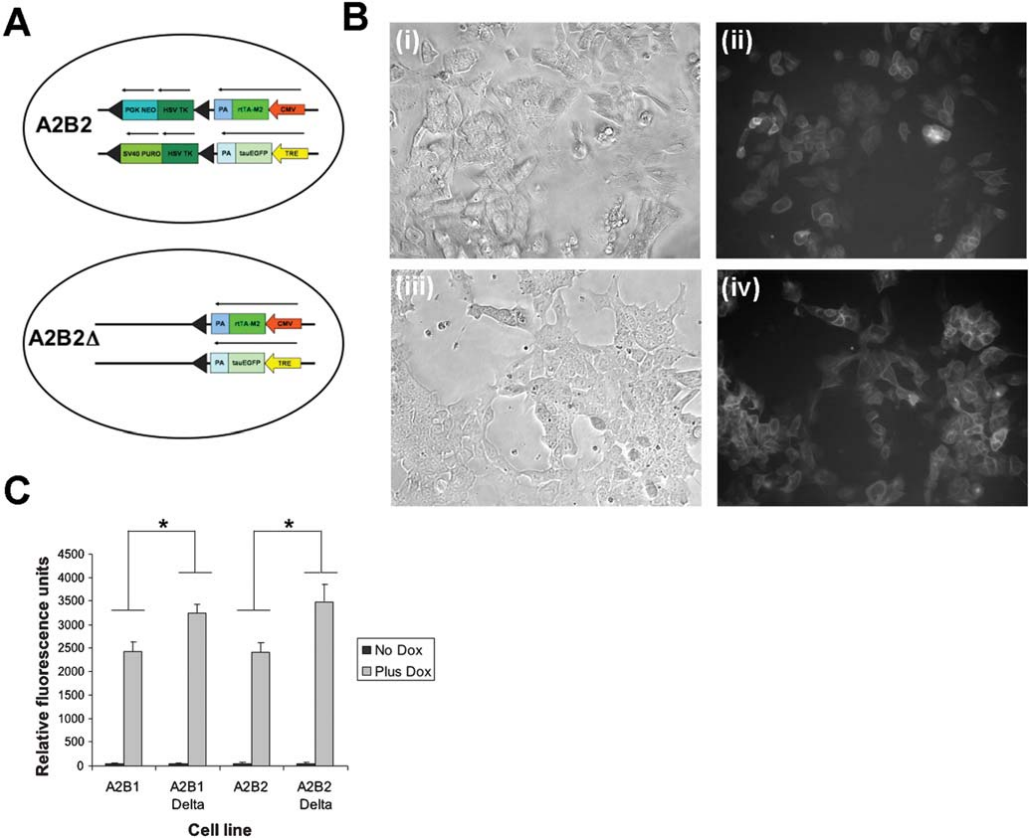
Expression level can be increased when selectable marker is removed. (**A**) Diagram of orientation of constructs in cell lines. (**B**) A2B2 and A2B2*Δ* cells induced with doxycycline (**i**) A2B2 phase contrast (**ii**) A2B2 EGFP expression (**iii**) A2B2*Δ* phase contrast (**iv**) A2B2*Δ* EGFP expression (**C**) Graph of expression levels in induced and uninduced cell lines quantitated by fluorimetry. Expression of EGFP is higher when these cell lines have lost the selectable marker cassette (*F*(1,40) = 77.25, *p*<0.0001). The orientation of the TRE EGFP makes no difference to expression level (*F*(1,40) = 1.14, *p* = 0.2910). Each point is an average of three measurements each from two independently targeted cell lines. Error bars denote standard deviations. Asterisks indicate statistically significant differences.

In order to test if the expression level was influenced by the sequences present in the selectable marker cassette, it was removed by Cre recombination. Recombination at the loxP sites flanking the marker gene removes the thymidine kinase expression cassette (Figure 4A). Cells were transiently transfected with a Cre expression plasmid and selected with gancyclovir. After selection in gancyclovir, resistant cell lines were analysed by PCR to ensure the selectable marker had been removed. More than 90% of the colonies recovered had undergone the appropriate recombination to remove the selectable marker (data not shown).

Resistant colonies were then analysed for EGFP expression by administration with doxycycline. For the cell lines A2B1*Δ* and A2B2*Δ*, with the selectable marker cassette removed, robust expression of EGFP was induced by addition of dox (Figure 4B). The expression in these cell lines is significantly higher than the expression of the cell lines before removal of the selectable marker (Figure 4C). This suggests that the sequences in the selectable marker had been affecting the expression of the EGFP. When these sequences are removed the EGFP expression level is increased.

### Expression level is decreased when selectable marker is removed and promoter is in same orientation as ROSA26 promoter

**Figure 5:**
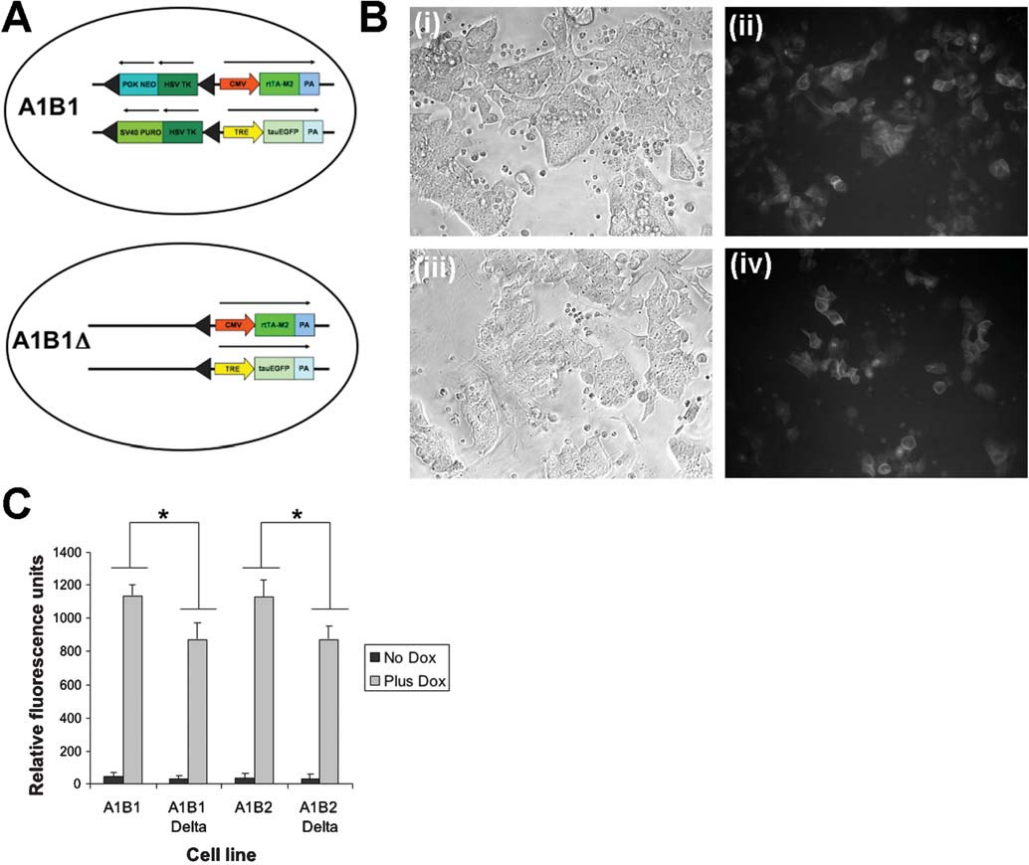
Expression level is decreased when selectable marker is removed and promoter is in same orientation as ROSA26 promoter. (**A**) Diagram of orientation of constructs in cell lines. (**B**) Α1Β1 ανδ Α1Β1Δ cells induced with doxycycline (**i**) A1B1 phase contrast (**ii**) A1B1 EGFP expression (**iii**) A1B1*Δ* phase contrast (**iv**) A1B1*Δ* EGFP expression (**C**) Graph of expression levels in induced and uninduced cell lines quantitated by fluorimetry. Expression of EGFP is lower when the selectable marker is removed from the A1 cell lines (*F*(1,40) = 51.77, *p*<0.001). Again the orientation of the TRE-EGFP makes no significant difference to the expression levels observed (*F*(1,40) = 0.05, *p* = 0.8295). Each point is an average of three measurements each from two independently targeted cell lines. Error bars denote standard deviations. Asterisks indicate statistically significant differences.

The selection marker was removed from the A1B1 and A1B2 cell lines by transient transfection of Cre recombinase and selection of the cells on gancyclovir (Figure 5A). Resistant cell lines were analysed by PCR to ensure that the selectable marker gene had been removed (data not shown).

Administration of dox to these cell lines again lead to a robust increase in the expression of EGFP (Figure 5B). However the expression level induced in the A1B1*Δ* and A1B2*Δ* cell lines without the selectable marker was lower than the cell lines. In this case it is likely that the selectable marker may have been insulating the CMV promoter from the effects of the endogenous ROSA26 promoter.

## Discussion

The ROSA26 locus is widely used as a locus for expressing transgene sequences. The data in this paper suggest that the sequences at the ROSA26 locus may have a significant effect of expression of sequences targeted to the locus. Transgene expression was seen to be highly dependent on orientation when inserted into the ROSA26 locus. The expression level is lower when the transgene is adjacent to, and in the same orientation as, the endogenous promoter. It seems likely that this may be caused by transcriptional interference with the endogenous ROSA26 promoter.

A recent report showed that the β-globin locus control region can silence as well as activate gene expression [28]. This silencing was shown to occur by transcriptional interference and was dependent on the orientation of the introduced transgene. This is a similar effect on gene expression as that observed here and it seems likely that a similar effect may be causing the orientation dependent transgene expression at the ROSA26 locus. Transcriptional interference has been proposed to influence gene expression on a genome wide basis [29]. And it has also been demonstrated that two transgenes will interfere with each other when targeted into the same locus [30]. This interference is more pronounced when the transgenes are arranged in the same orientation, similar to the arrangement of the A1 transactivator construct and the endogenous ROSA26 promoter. The A1 activator construct shows a lower level of expression than A2 (Figure 3C and D).

Deleting the selectable marker which is located between the endogenous ROSA26 promoter and the introduced CMV promoter seemed to enhance this effect. The A1B1*Δ* cell lines show higher expression that the A1B1 cells (Figure 5C). Removing the selectable marker, including both the HSV-tk and PGK-neo genes, brings the CMV promoter much closer to the endogenous promoter. This again implies that the reduced expression is dependent on the activity of the endogenous promoter and suggests that in this case the selectable marker may be partly acting as an insulator to reduce the effect of the ROSA26 promoter on transgene expression [31]. It has been found that suppression of downstream expression in tandem constructs is relieved when a polyadenylation and pause site separate the genes [32]. As the selectable marker contains two genes with polyadenylation sites this may explain why it acts as an insulator sequence in this case.

On the other hand removing the selectable marker when the CMV promoter is in the opposite orientation to the ROSA26 promoter enhances EGFP expression levels. The A2B2Δ cell lines show higher expression than the A2B2 cells (Figure 4C). This may be because the HSV-tk and PGK-neo genes in the selectable marker are interfering with the expression of the transgene and when removed this expression level is increased. It has been observed that insertion of a transgenic selectable marker to make a gene knockout can influence the expression of neighbouring genes [33]. Often dramatically different phenotypes can be observed in the presence and absence of a selectable marker. In this case removing the marker enhances the expression of the remaining A2 activator transgene.

The expression of EGFP is dependent on the expression of both the activator and responder constructs. The orientation of the activator transgene has a more significant effect on EGFP expression than the orientation of the responder. For example, the difference between the EGFP expression in the A2B2 and A1B2 is greater than the difference between the A1B1 and A1B2 (Figure 3B). Activation of EGFP expression must be more reliant on the level of the rtTA present in the cells prior to administration of dox. There is still a small effect of the orientation of the responder transgene.

It is not clear why the effect is more pronounced on the activator than the responder. It is possible that upon DNA binding in response to dox, the VP16 activation domain of the rtTA is strong enough to overcome local effects of the integration site. Hence EGFP levels which result from a combination of the expression of both transgenes, appear to depend more on the level of activity of the CMV promoter than the orientation of the of the responder element. The VP16 activation domain has been shown to be a potent transcriptional activator that can interact with many proteins, including transcription factors and chromatin-modifying co-activators, and can overcome the repressive effects of heterochromatin [34]–[36].

It is impossible to rule out that removing the selectable marker cassette leads to an increase in expression levels of the rtTA which is toxic to the cell. Substantial overexpression of the VP16 activator has been shown to be toxic to cells [37], [38]. However as the reverse orientation activates EGFP expression more efficiently and shows higher rtTA RNA expression levels, this possibility seems unlikely.

When the average expression levels of genetically identical clones are compared (*e.g*. two A2B2 cell lines), the variation between the cell lines is approximately 10–15%. This is not much higher than the variation between replicate expression level measurements of the same cell line. This suggests that targeting transgenes to the ROSA26 locus allows repeatable and reliable expression of inserted sequences.

It would be of interest to determine whether the changes in expression levels observed are due to changes in mosaic expression. Although there is variation in expression level between different cells, the majority of the undifferentiated ES cells express at least a small amount of the EGFP in all the cell lines examined. It seems more likely that the differences between cell lines are due to a change in expression levels within cells rather than a change in the proportion of cells which express the transgenes. A similar effect has been observed on the CMV promoter driven EGFP transgene in 293 cells. silencing of the transgene occurred by a reduction of expression levels rather that a decrease in the proportion of expressing cells [39]. This effect could be analysed in more detail by fluorescent activated cell sorting of the clones. Any analysis of this sort could be complicated by the fact there are likely to be different levels of differentiated cells in embryonic stem cell cultures and differentiated cells are likely to inactivate the CMV promoter [40].

The orientation dependent expression at the ROSA26 locus may have profound effects on expression on ES cells but also may effect expression in transgenic mice. Transcriptional interference has been proposed as a mechanism for transgene silencing [31]. It is possible therefore that although transcriptional interference does not completely silence transgene expression in ES cells that the difference in expression may be more dramatic in adult mouse tissues derived from the ES cells.

It may be possible that these effects could be avoided if the transgene was targeted upstream of the ROSA26 promoter. Indeed a similar strategy has been used to target exogenous sequences to the HPRT locus [14], [21]–[23]. In this case the expression level and pattern of the transgenes was not dependent on the orientation of the inserted sequences. However as the HPRT locus is on the X chromosome it will be randomly inactivated in female mice, which may not be ideal for every experiment.

This study has allowed us to define an optimal orientation for the introduction of exogenous promoters and transgene sequences into ROSA26. Targeting transgenes to a region of the genome, which allows optimal expression, will allow be significantly more efficient than the conventional pronuclear microinjection approach for introducing a large number of transgene constructs. As expression levels are unpredictable following pronuclear microinjection, multiple transgenic lines must be generated bred and analysed for expression. Targeting transgenes to a defined genomic region circumvents the necessity for multiple lines to be analysed for expression. This approach is therefore much more easily scalable that the conventional approach. The CMV promoter was used in the initial set of experiments as it sensitive to position effects in ES cells and although the results described apply only to this promoter, work is currently underway to test a range of neuron specific promoters to determine how efficiently these work when targeted to the ROSA26 locus.

Overall these results suggest that although targeting transgenes is an extremely useful tool in overcoming the gene silencing effects associated with the conventional transgenic approach, care must be take to avoid any adverse effect of the local chromatin structure and endogenous gene sequences on the inserted transgene.

## Materials and Methods

### Vector construction

Activator constructs were generated by cloning the CMV-rtTA-SV40 late polyA fragment from pUHrT62-1 [8] and the selectable marker from pLTNL [41], which includes both the PGK-neo and HSV-tk resistance genes flanked by loxP sites, were cloned into pROSA26-1(16). Responder constructs were generated by cloning EGFP fused to tau into a modified pTRE2Hyg (Clontech) and the fragment containing the tet operator and EGFP was subcloned into pROSA26-1 along with the selectable marker from pLTNL in which the PGK-Neo resistance gene had been swapped for an SV40-Puro-SV40 polyA derived from pPUR (Clontech). For electorporation 100 µg of plasmid DNA was linearised by digestion with SwaI and ethanol precipitated before resuspension in HBS.

### Embryonic stem cell culture

ES cells were maintained without feeders in Glasgow's modification of Eagle's medium (Invitrogen) supplemented with 400 U/ml recombinant murine leukaemia inhibitory factor (LIF; Chemicon), 0.1 mM MEM non-essential amino acids (Invitrogen), 10% foetal bovine serum Stem Cell Technologies) and 0.1 mM 2-mercaptoethanol (Sigma). HM-1 ES cells [42] were electroporated by mixing 1 x 10^7^ cells with 100 µg of linearised DNA (targeting experiments) in 0.8 ml of HEPES-buffered saline (pH 7.5), giving a single pulse at 800 V, 3 µF (Bio-Rad gene pulser) and plating at 1 x 10^6^ cells/10 cm dish. Selection was applied 20–24 h after electroporation. Cells were selected in either G418 (300 µgml^−1^), puromycin (1 µgml^−1^) or gancyclovir (1 µgml^−1^). EGFP was induced by addition of doxycycline usually (1 µgml^−1^) to normal growth medium.

### DNA analysis

Cells were lysed and DNA extracted by standard methods. Initial genotyping and screening for loss of the selectable marker was performed by PCR using Expand HiFi (Roche) according to the manufacturer's recommendations. Primers used for genotyping targeted ES cells were 5′ CGCCTAAAGAAGAGGCTGTG and 3′ GCCTGAAGAACGAGATCAGC. For screening for loss of selectable marker a different 3′ primer for each transgene was used in combination with the 5′ genotyping primer. (A1, GAGCGAGTTTCCTTGTCGTC; A2, ACGCTATCTGTGCCAAGGTCC; B1, TCCCGGTGTCTTCTATGGAG; B2, CCCAGTCATAGCTGTCCCTC)

For Southern blotting after EcoRI digestion, DNA was electrophoresed through 0.8% agarose and transferred onto Zetaprobe according to the manufacturer's recommendations (Bio-Rad). Hybridisation was performed at 65°C. Probes were labelled by random hexamer labelling (Rediprime II, Amersham) according to the manufacture's instructions. The final wash was with 0.1XSSC, 0.1% SDS at 65°C. Probes for the locus were isolated by PCR from wild-type genomic DNA using the folloing primers using standard conditions (5′ probe: TGGAGTAGGCAATACCCAGG and CACAGCCTCTTCTTTAGGCG, 3′ probe: GGCACTGTTCATTTGTGGTG and GTGCCTGTGGAGGCTAGAAG)

For reverse-transcriptase PCR (RT-PCR), total RNA was isolated from ES cell cultures using Qiagen RNeasy Mini Kit according to the manufacturer's instructions. First strand cDNA was made from RNA, using oligo dT primers, with RETROscript (Ambion Inc.) PCR was then performed on the cDNA using standard conditions with primers for rtTA2^s^-M2 (5′ CTGTGTCAGCAAGGCTTCTC and 3′ TCAGCAGGCAGCATATCAAG)

### Fluorimetry

EGFP expression was quantitated by fluorimetry as described by Yata *et al.* [43]. Briefly cells were harvested and homogenized in phosphate-buffered saline containing 0.5% Triton X100, 10 µgml^−1^ leupeptin, 20 µgml^−1^ aprotinin, and 0.1 µM phenylmethyl sulfonyl fluoride (PMSF). Homogenates were centrifuged in a microcentrifuge for 20 minutes, the supernatants collected, and protein concentrations determined using a protein assay reagent (BioRad).GFP fluorescence was assessed with a fluorometer (Turner Biosystems). Relative fluorescence units per mg of protein are shown on the graphs. Each point is an average of three measurements each from two independently targeted cell lines. Error bars denote the standard deviation calculated from these six measurements. Between-measures ANOVAs were conducted using Statview 5.0.1, as appropriate.
